# Preparative Method for Asymmetric Synthesis of (*S*)-2-Amino-4,4,4-trifluorobutanoic Acid

**DOI:** 10.3390/molecules24244521

**Published:** 2019-12-10

**Authors:** Jianlin Han, Ryosuke Takeda, Xinyi Liu, Hiroyuki Konno, Hidenori Abe, Takahiro Hiramatsu, Hiroki Moriwaki, Vadim A. Soloshonok

**Affiliations:** 1 College of Chemical Engineering, Nanjing Forestry University, Nanjing 210037, Jiangsu, China; hanjl@njfu.edu.cn (J.H.); kprxianchan@163.com (X.L.); 2 Hamari Chemicals Ltd., 1-4-29 Kunijima, Higashi-Yodogawa-ku, Osaka 533-0024, Japan; ryosuke-takeda@hamari.co.jp (R.T.); takahiro-hiramatsu@hamari.co.jp (T.H.); 3 Department of Biochemical Engineering, Graduate School of Science and Engineering, Yamagata University, Yonezawa, Yamagata 992‑8510, Japan; konno@yz.yamagata-u.ac.jp; 4 Department of Organic Chemistry I, Faculty of Chemistry, University of the Basque Country UPV/EHU, Paseo Manuel Lardizábal 3, 20018 San Sebastián, Spain; 5 IKERBASQUE, Basque Foundation for Science, María Díaz de Haro 3, Plaza Bizkaia, 48013 Bilbao, Spain

**Keywords:** Ni complex, fluorinated amino acid, large-scale synthesis, asymmetric synthesis, glycine Schiff base

## Abstract

Enantiomerically pure derivatives of 2-amino-4,4,4-trifluorobutanoic acid are in great demand as bioisostere of leucine moiety in the drug design. Here, we disclose a method specifically developed for large-scale (>150 g) preparation of the target (*S*)-*N*-Fmoc-2-amino-4,4,4-trifluorobutanoic acid. The method employs a recyclable chiral auxiliary to form the corresponding Ni(II) complex with glycine Schiff base, which is alkylated with CF_3_–CH_2_–I under basic conditions. The resultant alkylated Ni(II) complex is disassembled to reclaim the chiral auxiliary and 2-amino-4,4,4-trifluorobutanoic acid, which is in situ converted to the N-Fmoc derivative. The whole procedure was reproduced several times for consecutive preparation of over 300 g of the target (*S*)-*N*-Fmoc-2-amino-4,4,4-trifluorobutanoic acid.

## 1. Introduction

The modern paradigm in drug discovery is based on two major traits. The first includes mimicking the three-dimensional structure of the targeted protein receptor by incorporation of tailor-made amino acids (AAs) [1,2,3,4,5,6,7,8]. The second is to increase the metabolic stability of a drug molecule by the strategic fluorine for hydrogen substitution [9,10,11,12,13]. Obviously, the presence of a tailor-made AA’s backbone and fluorinated residues provide an additional host of subtle useful properties, allowing fine-tuning of the desired bio-activity and pharmacokinetics [14,15]. In this line of structural inquiry, fluorinated AAs, including α- [16,17,18,19,20,21,22,23,24] and β-derivatives [25,26,27], are considered of distinct potential in the modern drug design [28]. Synthesis and biological applications of fluorinated tailor-made AAs reported before 1994 are comprehensively covered by the book [29]. More recent data on the remarkable progress made in this area of research can be found in the reviews [16,17,18,19,20,21,22,23,24,25,26,27] and the current publications [30,31,32,33,34,35,36,37,38,39,40,41,42,43,44,45,46,47,48,49,50,51]. Synthesis of fluorinated AAs is part of a more general field of asymmetric synthesis of tailor-made amino acids. Therefore, some recent advances, covered by the excellent review articles, should be mentioned [52,53,54,55,56,57,58,59,60,61].

**Figure 1:**
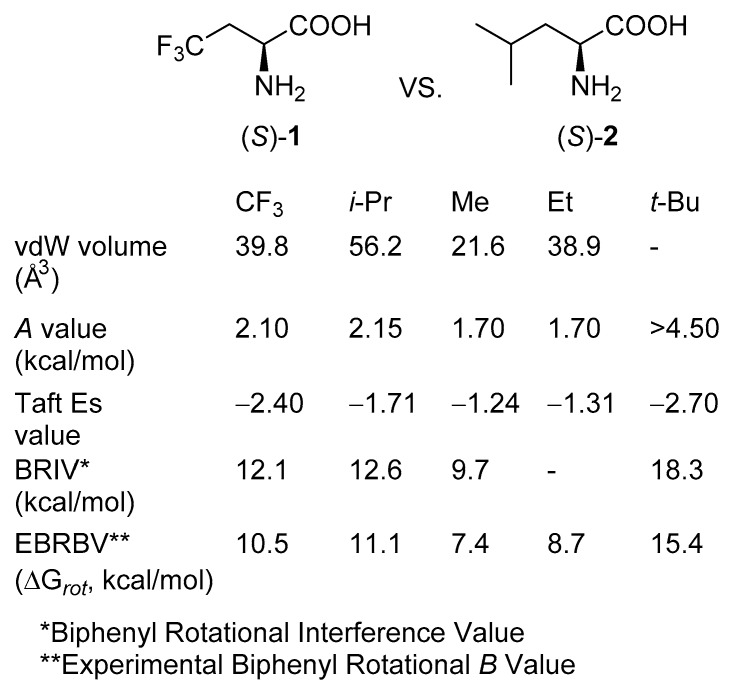
Bioisosteric relationships between CF_3_ and *i*-Pr groups in the context of 2-amino-4,4,4-trifluorobutanoic acid **1** substitution for leucine **2** in the drug design.

One of the most rapidly growing areas in the modern drug design is the application of fluorinated residues as bioisosteres of naturally occurring moieties [28]. In particular, based on considerations of the biologically relevant size, as cumulatively defined by van der Waals volume (vdW), A values, Taft Es values, and biphenyl interference values [62,63,64,65,66], trifluoromethyl group has often been considered to be isosteric with an iso-propyl substituent. Although a CF_3_ and *i*-Pr are clearly of a different shape, they are sterically much closer related than to Me, Et, or *t*-Bu groups. As presented in Figure 1, the differences projected by several biphenyl rotational values are relatively modest, suggesting steric mimicry between these two groups, though the effective functional mimicry is very much dependent upon the actual biochemical settings [67]. In this context, the bioisosteric relationships between CF_3_ and *i*-Pr can be used for 2-amino-4,4,4-trifluorobutanoic acid **1** [68,69,70,71,72,73,74,75,76] substitution for leucine **2** in the de novo design of biologically active peptides and peptidomimetics [77,78,79,80,81,82,83].

As part of our ongoing research program focused on the application of fluorinated tailor-made AAs in pharmaceutical discovery, we needed a convenient access to large quantities of Fmoc-2-amino-4,4,4-trifluorobutanoic acid **1** in both enantiomeric forms. Here, we disclose synthetic procedures specifically developed for large-scale preparation of the (*S*)-*N*-Fmoc-2-amino-4,4,4-trifluorobutanoic acid. The procedure was reliably reproduced several times for consecutive preparation of over 300 g of the target derivative of tailor-made AAs **1**.

## 2. Results and Discussion

**Scheme 1:**
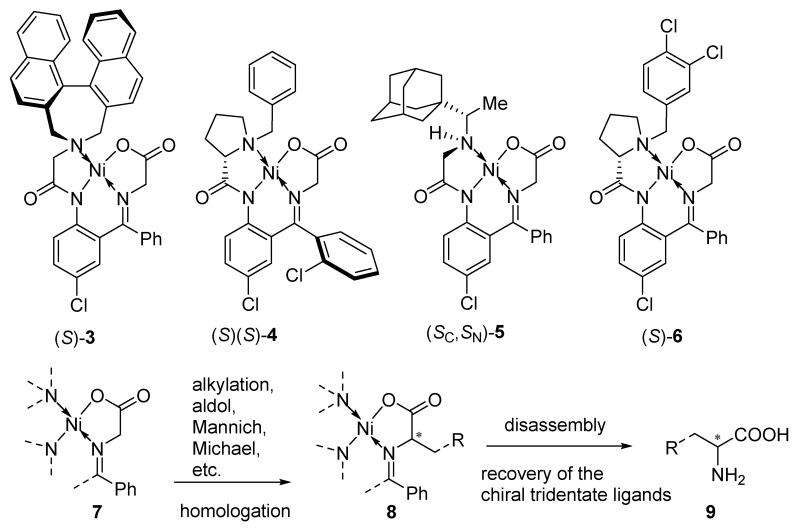
Four major types of chiral nucleophilic glycine equivalents **3**–**6** and their homologation to the target enantiomerically pure amino acids (AAs) **9** (carbon center with “*” means this is a carbon center).

Our interest in the structural types of tailor-made AAs includes phosphonic analogs [84,85,86], sterically constrained [87,88] and fluorine-containing AAs [89,90,91], all of which are of increasing importance in modern drug design [2,3,4,5,6,7,8]. Moreover, we carefully study nonlinear chiroptical phenomena, such as self-disproportionation of enantiomers [92,93,94] and its manifestation in the properties of AAs [95,96] and chiral marketed pharmaceuticals [97,98]. In the context of synthetic methodology, the chemistry of Ni(II) complexes of AA Schiff bases (Scheme 1) [99,100,101,102] has emerged as a dominant, most commonly used approach for asymmetric synthesis of tailor-made AAs. Using the modular approach for the design of chiral Ni(II) complexes of glycine Schiff bases [103,104], the recent research activity has been focusing around four structural types of chiral nucleophilic glycine equivalents **3**–**6** possessing elements of axial **3** [105], both axial and central **4** [106,107] chirality, carbon and nitrogen stereogenic centers **5** [108,109,110], and a proline-derived complex **6** bearing electron-deficient benzyl moiety [111,112]. The Ni(II) complex methodology can be used for direct dynamic kinetic resolution of racemic α- [113,114,115] and β-AAs [116], provided that the corresponding racemic AAs are readily commercially available. More general application of this chemistry is based on homologation of the glycine moiety 7 via, for example, alkylations [117,118], aldol [119,120], Mannich [121,122], and Michael [123,124,125] addition reactions. Multi-step transformations can also be realized, as demonstrated by efficient synthesis of (1*R*,2*S*)-1-amino-2-vinylcyclopropanecarboxylic acid [126,127]. Derivatives **8** are conveniently disassembled to release the target AAs **9**, along with recycling of the corresponding chiral ligands, rendering the process commercially attractive.

Chemical properties and particularities of the homologation of glycine Schiff base complexes **3**–**6** are still under investigation, but proline-derived (*S*)-**6** showed some advantageous reactivity and stereochemical outcome under alkyl halide alkylation conditions. Based on the previous data [68], we decided to use complex (*S*)-**6** for the large-scale asymmetric synthesis of 2-amino-4,4,4-trifluorobutanoic acid **1**. 

**Scheme 2:**
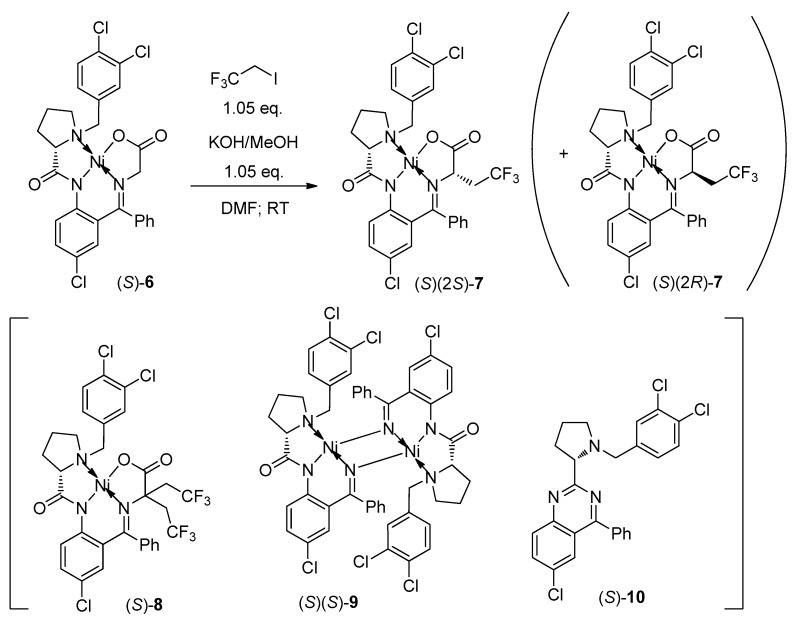
Alkylation of (*S*)-**6** with ICH_2_CF_3_; major diastereomer (*S*)(2*S*)-**7** and expected byproducts (*S*)(2*R*)-**7**, (*S*)-**8**, (*S*)-**9**, and (*S*)-**10**.

*Alkylation of (S)-**6** with ICH_2_CF_3_.* The alkylation of chiral glycine equivalent (*S*)-**6** with trifluoroethyl iodide is presented in Scheme 2. The development of a commercially viable, large-scale synthetic process is significantly more challenging as compared with a methodological investigation. Thus, besides the standard variables such as chemical yield and stereochemical outcome, special attention must be paid to operational convenience and overall cost structure. To optimize the alkylation step, we meticulously evaluated series of reaction solvents, bases, and stoichiometry of the reaction. For example, among the solvents promising results were obtained in DMSO, CH_3_CN, and DMF. The latter was eventually selected as the optimal solvent. Among the bases, we assessed KOH, NaOH, NaOMe, KOMe, KOH/MeOH, and NaOH/MeOH in various commercially available concentrations and combinations. As a result, solution of KOH in methanol was determined to be the optimal base. 

Normally, alkylation of glycine Schiff bases complexes with proline-derived ligands of type **6** is conducted in the presence of a large excess of a base, usually 5–10 equivalents [128]. Therefore, it was an important discovery that for large-scale synthesis, the optimized conditions required only insignificant 5 mol% excess of the base. The same unexpected stoichiometry was found to be optimal for the alkylating reagent. Thus, only 5 mol% excess of the trifluoroethyl iodide was sufficient to achieve optimal yield and the stereochemical outcome. 

As shown in Scheme 2, possible byproducts in this reactions include minor diastereomer (*S*)(2*R*)-**7** and bis-alkylated product (*S*)-**8**, which are typical impurities in the alkylation reactions of glycine Ni(II) complexes [129]. Other byproducts, such as binuclear complex (*S*)(*S*)-**9** and 4-phenylquinazoline (*S*)-**10**, are the results of oxidative degradation of starting (*S*)-**6** under the action of strong base in the presence of molecular oxygen [130,131]. These byproducts can be effectively eliminated by conducting the alkylation procedure under inert atmosphere. In the present case the reaction was conducted under N_2_. 

Another important discovery made in this work was the two-step quenching of the reaction mixture, allowing the precipitation the diastereomerically pure alkylation product (*S*)(2*S*)-**7**. Thus, despite the fact that alkylation of glycine complex (*S*)-**6** takes place with very high diastereoselectivity, usually >97% de, purification of the major diastereomer to diastereomerically pure state can be expensive in terms of time and silica gel and solvents. Therefore, for a large-scale synthesis, it was unquestionably needed to find a convenient and efficient procedure for isolation of the major diastereomer with maximum chemical yield. After extensive experimentation, we found that quenching the reaction mixture with calculated amount of water in specific sequence allows to solve this critical problem. In particular, the reaction mixture was first treated with water 3-times the amount of solvent DMF, allowing the mixture to be stirred at 20~40 °C for 1 h. This was followed by the second addition of water 2-times the original amount of DMF and stirring for 1 h at the same temperature. This treatment gave rise to the precipitation of major diastereomer (*S*)(2*S*)-**7** of greater than 99% de. Summary of the optimized conditions for the alkylation of (*S*)-**6** with ICH_2_CF_3_ is presented in Table 1.

As one can see from the Table 1, a quite acceptable chemical yield of alkylation product (*S*)(2*S*)-**7** (~80%) was obtained using essentially stoichiometric amounts of the reagents. Most importantly, the two-step quenching procedure allowed precipitation of virtually chemically and diastereomerically pure target product (*S*)(2*S*)-**7**. Minor diastereomer (*S*)(2*R*)-**7** and byproducts **8**–**10** could be detected in the aqueous washings but in concentrations below 1%. Some amounts (~5%) of target (*S*)(2*S*)-**7** were also detected in the aqueous phase, but their recovery deemed economically inefficient. Of particular importance was the confirmation of excellent reproducibility of this process. As presented in Table 1, the reaction was performed on 80 g scale (entry 1), and two times on 250 g scale (entries 2 and 3). In all cases, the chemical yield and stereochemical outcome were essentially the same, underscoring the reliability and practicality of this newly developed large-scale process. 

**Scheme 3:**
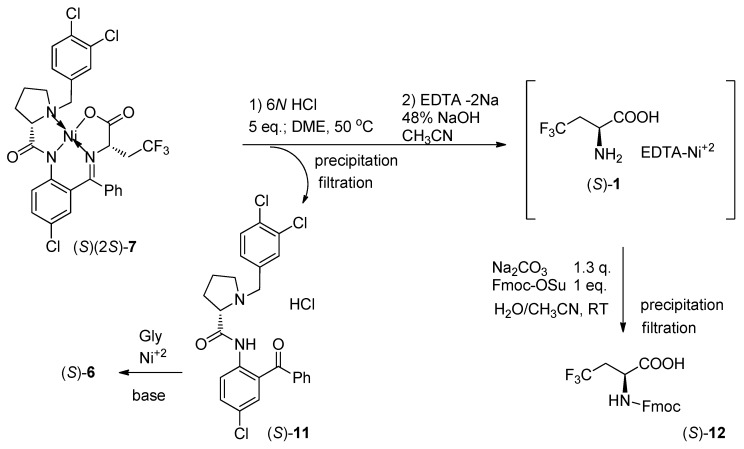
Disassembly of (*S*)(2*S*)-**7**, reclaiming chiral ligand (*S*)-**11**, and preparation of target AAs derivative (*S*)-**12**.

Disassembly of major diastereomer (*S*)(2*S*)-**7** and preparation of (*S*)-**12**. The procedure for disassembly of diastereomerically pure (*S*)(2*S*)-**7**, synthesis of (*S*)-**12** and the recovery of chiral ligand (*S*)-**11** is presented in Scheme 3. 

Generally, the disassembly of Ni(II) complexes of this type is conducted in methanol under the action of 3N hydrochloric acid [99,100,101,102]. However, under these conditions, some amounts of the corresponding methyl esters are being formed leading to some complications in isolation of the target products and lower chemical yields. For the present large-scale synthesis, we decided to use dimethoxyethane (DME), which is a rather chemically inert, low-cost industrial solvent. Another novelty in the present procedure was the use of 6N HCl, allowing to reduce the reaction time. Thus, heating the mixture of (*S*)(2*S*)-**7** and 6N HCl in DME at 50 °C for about 1 h resulted in evident color change form a bright-red to a green, indicating disassembly of the Ni(II) complex (bright-red) and formation of NiCl_2_ (green). The mixture was quenched with calculated amount of water, allowing precipitating hydrochloric salt of chiral ligand (*S*)-**11**. Recovery and reuse of the chiral ligand are usually more than 90%. The first crop of crystals (80%) is always >98% purity and can be reused without purification; the second crop (~20%) is ~95% pure and should be recrystallized again for reuse. Overall yield of the reclaimed ligand is usually ~95% and of >98% purity. Since the structure of the ligand is not changed, it can be reused indefinitely. Again, the loss is about 5% per cycle. The most expensive component of the whole procedure, simply by filtration, is the most practically attractive feature of this method, rendering the process commercially competitive. 

The filtrate, containing hydrochloric salt of amino acid (*S*)-**1** and NiCl_2_, was treated with ethylenediaminetetraacetic acid (EDTA) to chelate the Ni(II) ions, followed by the reaction with Fmoc-OSu under usual Fmoc-protection conditions. Target product (*S*)-**12** was isolated by precipitation with toluene form EtOAc solution. As presented in Table 2, this procedure was tested first on 10 g scale (entry 1) of compound (*S*)(2*S*)-**7** disassembly, and then successfully reproduced on large, 220 g scale. The obtained chemical yields of 75–80% are quite acceptable for a multi-step procedure, with the final product being collected simply by filtration.

## 3. Materials and Methods

### 3.1. General Methods

All reagents and solvents were used as received. Reactions were monitored by thin layer chromatography on Merck silica gel 60-F_254_-coated 0.25 mm plates, detected by UV. Flash chromatography was performed with the indicated solvents on silica gel (particle size 0.064–0.210 mm). Yields reported are for isolated, spectroscopically pure compounds. HPLC was performed on a SHIMADZU LC-2010CHT chromatography system (Kyoto, Japan) and a CLASS-VPTM analysis data system (Kyoto, Japan). ^1^H-NMR spectra were recorded on Brüker AVANCE III-400 spectrometer (Biospin, Switzerland). 

### 3.2. Alkylation of (S)-***6*** with ICH_2_CF_3_

#### 3.2.1. 80 g Scale Reaction

To a 2000 mL four-necked flask was added *N*,*N*-dimethyl-formamide (DMF) (720 mL) under nitrogen atmosphere. Stirring was continued for 30 min with nitrogen flow. Then, the Ni–glycine complex (*S*)-**6** (80.0 g, 1.0 eq.) together with DMF (20 mL), 1,1,1-trifluoro-2-iodoethane (29.32 g, 1.05 eq.) together with DMF (20 mL), and KOH (8.14 g, 1.05 eq.) in MeOH (72 mL) were added to the flask. The mixture was stirred at 20~35 °C under a nitrogen atmosphere for 1 h. After that, H_2_O (240 mL, 3 v vs. glycine complex (*S*)-**6**) was added into the mixture and stirred for 1 h at 20~35 °C and precipitate was formed gradually. Then, H_2_O (160 mL, 2 v vs. glycine complex (*S*)-**6**) was added and stirred at the same temperature for 1.5 h. The precipitate was filtered and washed with H_2_O (160 mL, 2 v vs. glycine complex (*S*)-**6**), then dried by air for 18 h to give the product (*S*)(2*S*)-**7** (73.7 g, 81.1% yield, >99% de).

#### 3.2.2. 250 g Scale Reaction

To a 5000 mL four-necked flask, was added *N*,*N*-dimethyl-formamide (DMF) (2250 mL) under nitrogen atmosphere. Stirring was continued for 50 min with nitrogen flow. Then, the Ni–glycine complex (*S*)-**6** (250.0 g, 1.0 eq.) together with DMF (100 mL), 1,1,1-trifluoro-2-iodoethane (91.61 g, 1.05 eq.) together with DMF (50 mL), and KOH (25.42 g, 1.05 eq.) in MeOH (225 mL) together with DMF (100 mL) were added to the flask. The mixture was stirred at 20~35 °C under a nitrogen atmosphere for 1 h. After that, H_2_O (750 mL, 3 v vs. glycine complex (*S*)-**6**) was added into the mixture and stirred for 1 h at 20~40 °C and precipitate was formed gradually. Then, H_2_O (500 mL, 2 v vs. glycine complex (*S*)-**6**) was added and stirred at the same temperature for 1 h. The precipitate was filtered and washed with H_2_O (500 mL, 2 v vs. glycine complex (*S*)-**6**), then dried by air for 19.5 h to give the product (*S*)(2*S*)-**7** (228.50 g, 80.4% yield, 97.2 area%, >99% de).

### 3.3. Disassembly of Major Diastereomer (S)(2S)-***7*** and Preparation of (S)-***12***

To a 2000 mL four-necked flask was added dimethoxyethane (DME) (400 mL), Ni complex (*S*,2*S*)-**7** (220 g, 1.0 eq.) together with DME (30 mL) and HCl (6N, 268 mL, 5.0 eq.) together with DME (10 mL). The mixture was heated to 40~50 °C and stirred at this temperature for 1 h. After that, the solution was changed to a green suspension and was cooled to 20~40 °C. H_2_O (440 mL, 2 v) was added and the mixture was stirred at 20~40 °C for 1.5 h. The precipitate was filtered and washed with DME (88 mL, 0.4 v), 6N HCl (59 mL, 0.27 v), H_2_O (293 mL, 1.33 v), then H_2_O (220 mL, 1 v). The green filtrate was collected to give a solution of (*S*)-**1** (ca. 1500 mL).

To a 3000 mL 4-necked flask containing the above green filtrate was added ethylenediaminetetraacetic acid disodium salt hydrate (122.2 g, 1.02 eq.). With stirring, 48% NaOH (138 g, 5.2 eq.), Na_2_CO_3_ (1.3 eq., 44.35 g), Fmoc-OSu (1 eq., 108.57 g), and acetonitrile (200 mL, 4 v) were added. The resulted mixture was stirred at room temperature for 4 h and acetonitrile was removed. Then, EtOAc (180 mL) and 6N HCl (70 mL, 4 eq.) was added and the phases were separated. The water layer was extracted with ethyl acetate (100 mL), and the combined organic layer was washed with water (100 mL). The combined organic solution was dried with Na_2_SO_4_ (30 g), and then the filtrate was concentrated to 400 mL. The resulted solution was heated to 50~60 °C, and toluene (400 mL) was added. After that, it was concentrated until 400 mL, and EtOAc (100 mL) was added. Finally, toluene (400 mL) was added and then the solution was concentrated, followed by addition of additional toluene (400 mL) until 800 mL with stirring slowly at 20~35 °C. The precipitate was filtered, washed with toluene (160 mL), and dried in vacuo at 50 °C for 19 h to afford (*S*)-**12** (99.5 g, 81.5% yield 98.4% ee, a white powder).

^1^H-NMR (400 MHz, CD_3_OD): δ = 7.78 (d, *J* = 7.7 Hz, 2H), 7.69 (d, *J* = 7.7 Hz, 2H), 7.34−7.41 (m, 2H), 7.25−7.33 (m, 2H), 4.42−4.53 (m, 1H), 4.29−4.37 (m, 2H), 4.20−4.29 (m, 1H), 2.78−2.91 (m, 1H), 2.60−2.72 (m, 1H) (see Supplementary Materials).

## 4. Conclusions

The data disclosed in this paper demonstrate that alkylation of Ni(II) complex of glycine Schiff base with CF_3_–CH_2_–I can be successfully conducted on a scale of over 200 g of the corresponding complex, providing a reliable assess to enantiomerically pure (>99% ee) derivatives of (*S*)-2-amino-4,4,4-trifluorobutanoic acid. The developed method features recyclable chiral auxiliary and optimized operationally convenient conditions, rendering the reported procedure inexpensive and commercially viable. The broader importance of these results is a convincing evidence that the Ni(II) complexes methodology has a degree of practicality, deserving more detailed investigation for large-scale asymmetric synthesis of various pharmacologically valuable tailor-made amino acids.

## Figures and Tables

**Table 1:** Optimized conditions for the alkylation step and reproducibility of the method.

Entry	Scale	Conditions	Results
1	(*S*)-**6** 80 g	ICH_2_CF_3_ (1.05 eq. 29.32 g), KOH (96.8% assay, 1.05 eq. 8.14 g), MeOH (0.9 v, 72 mL), DMF (10 v, 800 mL), rt (room temperature), 1 h	(*S*)(2*S*)-**7** 73.7 g, 81.1% yield, >99% de (diasteromeric excess)
2	(*S*)-**6** 250 g	ICH_2_CF_3_ (1.05 eq. 91.61 g), KOH (96.8% assay, 1.05 eq. 25.42 g), MeOH (0.9 v, 225 mL), DMF (10 v, 2500 mL), rt, 1 h	(*S*)(2*S*)-**7** 229.1 g, 80.7% yield, 99.9% de
3	(*S*)-**6** 250 g	ICH_2_CF_3_ (1.05 eq. 91.61 g), KOH (96.8% assay, 1.05 eq. 25.42 g), MeOH (0.9 v, 225 mL), DMF (10 v, 2500 mL), rt, 1 h	(*S*)(2*S*)-**7** 228.5 g, 80.4% yield, >99% de

**Table 2:** Optimized conditions for preparation of Fmoc derivative (*S*)-**12** and reproducibility of the method.

Entry	(*S*)(2*S*)-7	Conditions	(*S*)-12
1	10 g	6N HCl (5 eq. 12.2 mL), DME (2 v, 20 mL), 50 °C, 1 h added H_2_O (2 v, 20 mL), 30 °C, 2 h then EDTA•2Na (1.02 eq. 5.55 g), 48% NaOH (4.2 eq. 5.1 g) Na_2_CO_3_ (1.3 eq. 2.02 g), Fmoc-OSu (1 eq. 4.94 g) MeCN (4 v, 9.2 mL), rt, 3 h	4.26 g, 76.7% yield 99.0% ee (enantiomeric excess)
2	220 g	6N HCl (5 eq. 268 mL), DME (2 v, 440 mL), 50 °C, 1 h added H_2_O (2 v, 440 mL), 30 °C, 2 h then EDTA•2Na (1.02 eq. 122.2 g), 48% NaOH (5.4 eq. 143.6 g) Na_2_CO_3_ (1.3 eq. 44.35 g), Fmoc-OSu (1 eq. 108.57 g) MeCN (4 v, 200 mL), rt, 4 h	92.7 g, 75.9% yield 98.8% ee
3	220 g	6N HCl (5 eq. 268 mL), DME (2 v, 440 mL), 50 °C, 1 h added H_2_O (2 v, 440 mL), 30 °C, 1.5 h then EDTA•2Na (1.02 eq. 122.2 g), 48% NaOH (5.2 eq. 138 g) Na_2_CO_3_ (1.3 eq. 44.35 g), Fmoc-OSu (1 eq. 108.57 g) MeCN (4 v, 200 mL), rt, 4 h	99.5 g, 81.5% yield 98.4% ee

## Supplementary Materials

The following are available online: NMR spectra.

## References

[1] Soloshonok V.A., Cai C., Hruby V.J., Meervelt L.V. (1999). Asymmetric Synthesis of Novel Highly Sterically Constrained (2*S*,3*S*)-3-Methyl-3-Trifluoromethyl- and (2*S*,3*S*,4*R*)-3-Trifluoromethyl-4-Methylpyroglutamic Acids. Tetrahedron.

[2] Henninot A., Collins J.C., Nuss J.M. (2018). The current state of peptide drug discovery: Back to the future?. J. Med. Chem..

[3] Blaskovich M.A.T. (2016). Unusual amino acids in medicinal chemistry. J. Med. Chem..

[4] Ma J.S. (2003). Unnatural amino acids in drug discovery. Chim. Oggi.

[5] Hodgson D.R.W., Sanderson J.M. (2004). The synthesis of peptides and proteins containing non-natural amino acids. Chem. Soc. Rev..

[6] Sato T., Izawa K., Aceña J.L., Liu H., Soloshonok V.A. (2016). Tailor-Made α-Amino Acids in Pharmaceutical Industry: Synthetic Approaches to (1*R*,2*S*)-1-Amino-2-vinylcyclopropane-1-carboxylic Acid (Vinyl-ACCA). Eur. J. Org. Chem..

[7] Wang S., Wang Y., Wang J., Sato T., Izawa K., Soloshonok V.A., Liu H. (2017). The second-generation of highly potent hepatitis C virus (HCV) NS3/4A protease inhibitors: Evolutionary design based on tailor-made amino acids, synthesis and major features of bio-activity. Curr. Pharm. Des..

[8] Soloshonok V.A., Izawa K. (2009). Asymmetric Synthesis and Application of alpha-Amino Acids.

[9] Mei H., Han J., Fustero S., Medio-Simon M., Sedgwick D.M., Santi C., Ruzziconi R., Soloshonok V.A. (2019). Fluorine-Containing Drugs Approved by the FDA in 2018. Chem. Eur. J..

[10] Zhu W., Wang J., Wang S., Gu Z., Aceña J.L., Izawa K., Liu H., Soloshonok V.A. (2014). Recent advances in the trifluoromethylation methodology and new CF3-containing drugs. J. Fluor. Chem..

[11] Zhu Y., Han J.L., Wang J., Shibata N., Sodeoka M., Soloshonok V.A., Coelho J.A.S., Toste F.D. (2018). Modern Approaches for Asymmetric Construction of Carbon–Fluorine Quaternary Stereogenic Centers: Synthetic Challenges and Pharmaceutical Needs. Chem. Rev..

[12] Tao J.Y., Song W.T., Sun L., Li Z.Z., Fu B., Cai Z.C., Xu H.J. (2019). Synthesis, Photo-physical Properties and Cell Imaging of Meso-2,6-dichlorophenyl Boron-dipyrromethene Derivatives. Chin. J. Struct. Chem..

[13] Yang J., Fan Y., Cai F., Xu X., Fu B., Wang S., Shen Z., Tian J., Xu H. (2019). BODIPY derivatives bearing borneol moieties: Enhancing cell membrane permeability for living cell imaging. Dyes Pigment..

[14] Zhou Y., Wang J., Gu Z., Wang S., Zhu W., Aceña J.L., Soloshonok V.A., Izawa K., Liu H. (2016). Next Generation of Fluorine-Containing Pharmaceuticals, Compounds Currently in Phase II–III Clinical Trials of Major Pharmaceutical Companies: New Structural Trends and Therapeutic Areas. Chem. Rev..

[15] Wang J., Sánchez-Roselló M., Aceña J.L., del Pozo C., Sorochinsky A.E., Fustero S., Soloshonok V.A., Liu H. (2014). Fluorine in Pharmaceutical Industry: Fluorine-Containing Drugs Introduced to the Market in the Last Decade (2001–2011). Chem. Rev..

[16] Smits R., Cadicamo C.D., Burger K., Koksch B. (2008). Synthetic strategies to α-trifluoromethyl and α-difluoromethyl substituted α-amino acids. Chem. Soc. Rev..

[17] Kukhar V.P., Sorochinsky A.E., Soloshonok V.A. (2009). Practical synthesis of fluorine-containing α-and β-amino acids: Recipes from Kiev, Ukraine. Future Med. Chem..

[18] Sorochinsky A.E., Soloshonok V.A. (2010). Asymmetric synthesis of fluorine-containing amines, amino alcohols, α- and β-amino acids mediated by chiral sulfinyl group. J. Fluor. Chem..

[19] Tarui A., Sato K., Omote M., Kumadaki I., Ando A. (2010). Stereoselective synthesis of α-fluorinated amino acid derivatives. Adv. Synth. Catal..

[20] Czekelius C., Tzschucke C.C. (2010). Synthesis of halogenated carboxylic acids and amino acids. Synthesis.

[21] Qiu X.L., Qing F.L. (2011). Recent advances in the synthesis of fluorinated amino acids. Eur. J. Org. Chem..

[22] Turcheniuk K.V., Kukhar V.P., Roeschenthaler G.V., Acena J.L., Soloshonok V.A., Sorochinsky A.E. (2013). Recent advances in the synthesis of fluorinated aminophosphonates and aminophosphonic acids. RSC Adv..

[23] Aceña J.L., Sorochinsky A.E., Soloshonok V.A. (2012). Recent advances in asymmetric synthesis of α-(trifluoromethyl)-containing α-amino acids. Synthesis.

[24] Aceña J.L., Sorochinsky A.E., Moriwaki H., Sato T., Soloshonok V.A. (2013). Synthesis of fluorine-containing α-amino acids in enantiomerically pure form via homologation of Ni(II) complexes of glycine and alanine Schiff bases. J. Fluor. Chem..

[25] Mikami K., Fustero S., Sánchez-Roselló M., Aceña J.L., Soloshonok V.A., Sorochinsky A.E. (2011). Synthesis of Fluorine Containing β-Amino Acids. Synthesis.

[26] Han J., Sorochinsky A.E., Ono T., Soloshonok V.A. (2011). Biomimetic Transamination—A Metal-Free Alternative to the Reductive Amination. Application for Generalized Preparation of Fluorine-Containing Amines and Amino Acids. Curr. Org. Synth..

[27] Soloshonok V.A., Kirilenko A.G., Fokina N.A., Shishkina I.P., Galushko S.V., Kukhar V.P., Svedas V.K., Kozlova E.V. (1994). Biocatalytic Resolution of β-Fluoroalkyl-β-Amino Acids. Tetrahedron.

[28] Meanwell N.A. (2018). Fluorine and Fluorinated Motifs in the Design and Application of Bioisosteres for Drug Design. J. Med. Chem..

[29] Kukhar V.P., Soloshonok V.A. (1994). Fluorine-Containing Amino Acids: Synthesis and Properties.

[30] Hao J., Milcent T., Retailleau P., Soloshonok V.A., Ongeri S., Crousse B. (2018). Asymmetric Synthesis of Cyclic Fluorinated Amino Acids. Eur. J. Org. Chem..

[31] Peng Y.Y., Liu P., Liu Z.J., Liu J.T., Mao H.F., Yao Y.L. (2018). Regio- and diastereoselective Reformatsky reaction of chiral fluoroalkyl α,β-unsaturated N-tert-butanesulfinyl ketimines: Efficient asymmetric synthesis of β-fluoroalkyl β-vinyl β-amino esters. Tetrahedron.

[32] Kondratov I.S., Bugera M.Y., Tolmachova N.A., Daniliuc C.G., Haufe G. (2018). Straightforward synthesis of fluorinated amino acids by Michael addition of ethyl bromodifluoroacetate to α,β-unsaturated α-amino acid derivatives. J. Fluor. Chem..

[33] Oliver M., Gadais C., Garcia-Pindado J., Teixido M., Lensen N., Chaume G., Brigaud T. (2018). Trifluoromethylated proline analogues as efficient tools to enhance the hydrophobicity and to promote passive diffusion transport of the L-prolyl-L-leucyl glycinamide (PLG) tripeptide. RSC Adv..

[34] Makki M.S.T., Reda M., Alharbi A.S. (2018). Synthetic Approach for Novel Fluorine Substituted α-Aminophosphonic Acids Containing 1,2,4-Triazin-5-One Moiety as Antioxidant Agents. Int. J. Org. Chem..

[35] Zhao J.B., Ren X., Zheng B.Q., Ji J., Qiu Z.B., Li Y. (2018). A diastereoselective Mannich reaction of α-fluoroketones with ketimines: Construction of β-fluoroamine motifs with vicinal tetrasubstituted stereocenters. Tetrahedron Lett..

[36] Bucci R., Das P., Iannuzzi F., Feligioni M., Gandolfi R., Gelmi M.L., Reches M., Pellegrino S. (2017). Self-assembly of an amphipathic ααβ-tripeptide into cationic spherical particles for intracellular delivery. Org. Biomol. Chem..

[37] Betts H.M., Milicevic Sephton S., Tong C., Awais R.O., Hill P.J., Perkins A.C., Aigbirhio F.I. (2016). Synthesis, in vitro evaluation, and radiolabeling of fluorinated puromycin analogues: Potential candidates for PET imaging of protein synthesis. J. Med. Chem..

[38] Bouhlel A., Alyami W., Li A., Yuan L., Rich K., McConathy J. (2016). Effect of α-Methyl versus α-Hydrogen Substitution on Brain Availability and Tumor Imaging Properties of Heptanoic [F-18] Fluoroalkyl Amino Acids for Positron Emission Tomography (PET). J. Med. Chem..

[39] Ayoup M.S., Cordes D.B., Slawin A.M.Z., O’Hagan D. (2015). Fluorine containing amino acids: Synthesis and peptide coupling of amino acids containing the all-cis tetrafluorocyclohexyl motif. Org. Biomol. Chem..

[40] Bandak D., Babii O., Vasiuta R., Komarov I.V., Mykhailiuk P.K. (2015). Design and synthesis of novel 19F-amino acid: A promising 19F NMR label for peptide studies. Org. Lett..

[41] Usuki Y., Wakamatsu Y., Yabu M., Iio H. (2014). A New Access to Fluorine-containing Asparagine and Glutamine Analogues via Pd-catalyzed Formate Reduction. Asian J. Org. Chem..

[42] Tkachenko A.N., Mykhailiuk P.K., Radchenko D.S., Babii O., Afonin S., Ulrich A.S., Komarov I.V. (2014). Design and Synthesis of a Monofluoro-Substituted Aromatic Amino Acid as a Conformationally Restricted 19F NMR Label for Membrane-Bound Peptides. Eur. J. Org. Chem..

[43] Shibata N., Nishimine T., Shibata N., Tokunaga E., Kawada K., Kagawa T., Sorochinsky A.E., Soloshonok V.A. (2012). Organic base-catalyzed stereodivergent synthesis of (*R*)- and (*S*)-3-amino-4,4,4-trifluorobutanoic acids. Chem. Commun..

[44] Shibata N., Nishimine T., Shibata N., Tokunaga E., Kawada K., Kagawa T., Aceña J.L., Sorochinsky A.E., Soloshonok V.A. (2014). Asymmetric Mannich reaction between (*S*)-*N*-(tertbutanesulfinyl)-3,3,3-trifluoroacetaldimine and malonic acid derivatives. Stereodivergent synthesis of (*R*)- and (*S*)-3-amino-4,4,4-trifluorobutanoic acids. Org. Biomol. Chem..

[45] Kiss L., Fueloep F. (2018). Selective Synthesis of Fluorine-Containing Cyclic β-Amino Acid Scaffolds. Chem. Rec..

[46] Milcent T., Hao J., Kawada K., Soloshonok V.A., Ongeri S., Crousse B. (2014). Highly Stereoselective aza-Baylis–Hillman Reactions of CF3-Sulfinylimines: Straightforward Access to α-Methylene β-CF3 β-Amino Acids. Eur. J. Org. Chem..

[47] Drouet F., Noisier A.F.M., Harris C.S., Furkert D.P., Brimble M.A. (2014). A Convenient Method for the Asymmetric Synthesis of Fluorinated α-Amino Acids from Alcohols. Eur. J. Org. Chem..

[48] Mei H., Hiramatsu T., Takeda R., Moriwaki H., Abe H., Han J., Soloshonok V.A. (2019). Expedient Asymmetric Synthesis of (*S*)-2-Amino-4,4,4-trifluorobutanoic Acid via Alkylation of Chiral Nucleophilic Glycine Equivalent. Org. Process Res. Dev..

[49] Mei H., Yin Z., Miwa T., Moriwaki H., Abe H., Han J., Soloshonok V.A. (2019). Convenient Asymmetric Synthesis of Fmoc-(*S*)-6,6,6-trifluoro-Norleucine. Symmetry.

[50] Yin Z., Moriwaki H., Abe H., Miwa T., Han J., Soloshonok V.A. (2019). Large-Scale Asymmetric Synthesis of Fmoc-(*S*)-2-Amino-6,6,6-Trifluorohexanoic Acid. ChemistryOpen.

[51] Mei H., Han J.L., Takeda R., Sakamoto T., Miwa T., Minamitsuji Y., Moriwaki H., Abe H., Soloshonok V.A. (2019). Practical Method for Preparation of (S)‑2-Amino-5,5,5-trifluoropentanoic Acid via Dynamic Kinetic Resolution. ACS Omega.

[52] Kim Y., Park J., Kim M.J. (2011). Dynamic kinetic resolution of amines and amino acids by enzyme–metal cocatalysis. ChemCatChem.

[53] Wang J., Zhang L., Jiang H., Chen K., Liu H. (2011). Application of nickel (II) complexes to the efficient synthesis of α- or β-amino acids. Chimia.

[54] Popkov A., De Spiegeleer B. (2012). Chiral nickel (II) complexes in the preparation of ^11^C-and ^18^F-labelled enantiomerically pure α-amino acids. Dalton Trans..

[55] So S.M., Kim H., Mui L., Chin J. (2012). Mimicking nature to make unnatural amino acids and chiral diamines. Eur. J. Org. Chem..

[56] D’Arrigo P., Cerioli L., Servi S., Viani F., Tessaroa D. (2012). Synergy between catalysts: Enzymes and bases. DKR of non-natural amino acids derivatives. Cat. Sci. Technol..

[57] D’Arrigo P., Cerioli L., Fiorati A., Servi S., Viani F., Tessaro D. (2012). Naphthyl-l-α-amino acids via chemo-enzymatic dynamic kinetic resolution. Tetrahedron.

[58] Periasamy M., Gurubrahamam R., Sanjeevakumar N., Dalai M., Alakonda L., Reddy P.O., Suresh S., Satishkumar S., Padmaja M., Reddy M.N. (2013). Convenient methods for the synthesis of chiral amino alcohols and amines. Chimia.

[59] Bera K., Namboothiri I. (2014). Asymmetric synthesis of quaternary α-amino acids and their phosphonate analogues. Asian J. Org. Chem..

[60] Metz A.E., Kozlowski M.C. (2015). Recent advances in asymmetric catalytic methods for the formation of acyclic α, α-disubstituted α-amino acids. J. Org. Chem..

[61] He G., Wang B., Nack W.A., Chen G. (2016). Syntheses and transformations of α-amino acids via palladium-catalyzed auxiliary-directed sp3 C–H functionalization. Acc. Chem. Res..

[62] Bott F., Field L.D., Sternhell S. (1980). Steric effects. A study of a rationally designed system. J. Am. Chem. Soc..

[63] Lunazzi L., Mancinelli M., Mazzanti A., Lepri S., Ruzziconi R., Schlosser M. (2012). Rotational barriers of biphenyls having heavy heteroatoms as ortho substituents: Experimental and theoretical determination of steric effects. Org. Biomol. Chem..

[64] de Riggi I., Virgili A., de Moragas M., Jaime C. (1995). Restricted rotation and NOE transfer: A conformational study of some substituted (9-anthry1)carbinol derivatives. J. Org. Chem..

[65] Belot V., Farran D., Jean M., Albalat M., Vanthuyne N., Roussel C. (2017). Steric scale of common substituents from rotational barriers of *N*-(o-substituted aryl)thiazoline-2-thione atropisomers. J. Org. Chem..

[66] Soloshonok V.A., Kukhar V.P., Galushko S.V., Svistunova N.Y., Avilov D.V., Kuzmina N.A., Raevski N.I., Struchkov Y.T., Pisarevsky A.P., Belokon Y.N. (1993). General Method for the Synthesis of Enantiomerically Pure β-Hydroxy-α-Amino Acids, Containing Fluorine Atoms in the Side Chains. Case of Stereochemical Distinction Between Methyl and Trifluoromethyl Groups. X-Ray Crystal and Molecular Structure of the Nickel(II) Complex of (2*S*,3*S*)-2-(Trifluoromethyl)threonine. J. Chem. Soc. Perkin Trans..

[67] Jagodzinska M., Huguenot F., Candiani G., Zanda M. (2009). Assessing the bioisosterism of the trifluoromethyl group with a protease probe. ChemMedChem.

[68] Walborsky H.M., Baum M.E. (1956). Chemical Effects of the Trifluoromethyl Group: III. Synthesis of 2-Amino-4,4,4-trifluorobutyric Acid. J. Org. Chem..

[69] Steglich W., Heininger H.U., Dworschak H., Weygand F. (1967). A General Method for the Preparation of β-Perfluoroalkylalanines. Angew. Chem. Int. Ed. Engl..

[70] Tsushima T., Kawada K., Ishihara S., Shiratori O., Higaki J., Hirata M. (1988). Fluorine containing amino acids and their derivatives. 7. Synthesis and antitumor activity of α- and γ-substituted methotrexate analogs. Tetrahedron.

[71] Seebach D., Buerger H.M., Schickli C.P. (1991). Stereoselektive Umsetzungen von rac-, (*R*), oder (*S*)-5-Alkyliden-2-t-butyl-3-methyl-4-oxo-1-imidazolidincarbonsäure-t-butylestern (chirale 2,3-Dehydroaminosäure-Derivate) und Herstellung einiger nichtproteinogener Aminosäuren. Ann. Chem..

[72] Schedel H., Dmowski W., Burger K. (2000). New Stereoconservative Syntheses of β,β,β- and γ,γ,γ-Trifluoro-α-amino, α-Hydroxy, and α-Mercapto Acids and Their Incorporation into a Peptide and Depsipeptide Fragment. Synthesis.

[73] Kondratov I.S., Logvinenko I.G., Tolmachova N.A., Morev R.N., Kliachyna M.A., Clausen F., Daniliuc C.G., Haufe G. (2017). Synthesis and physical chemical properties of 2-amino-4-(trifluoromethoxy) butanoic acid–α CF3O-containing analogue of natural lipophilic amino acids. Org. Biomol. Chem..

[74] Xin B.T., Huber E.M., de Bruin G., Heinemeyer W., Maurits E., Espinal C., Du Y., Janssens M., Weyburne E.S., Kisselev A.F. (2019). Structure-Based Design of Inhibitors Selective for Human Proteasome β2c or β2i Subunits. J. Med. Chem..

[75] Franko N., Grammatoglou K., Campanini B., Costantino G., Jirgensons A., Mozzarelli A. (2018). Inhibition of O-acetylserine sulfhydrylase by fluoroalanine derivatives. J. Enzyme Inhib. Med. Chem..

[76] Gambini L., Salem A.F., Udompholkul P., Tan X.F., Baggio C., Shah N., Aronson A., Song J., Pellecchia M. (2018). Structure-Based Design of Novel EphA2 Agonistic Agents with Nanomolar Affinity in Vitro and in Cell. ACS Chem. Biol..

[77] Papaioannou N., Fink S.J., Miller T.A., Shipps G.W., Travins J.M., Ehmann D.E., Rae A., Ellard J.M. (2019). Preparation of Substituted Imidazopyridines as Inhibitors of Plasma Kallikrein. Patent.

[78] Bilcer G.M., Kelly T.A. (2019). Preparation of Hexahydro(di)azepino[3,2,1-hi]indole-, Tetrahydro-7H-6-oxa-9a-Azabenzo[cd]azulene- and Tetrahydro-7H-6-thia-9a-azabenzo[cd]azulene-Containing Peptides as Granzyme B Directed Imaging and Therapy. Patent.

[79] Boss C., Cren S., Kimmerlin T., Lotz-Jenne C., Pothier J., Tidten-Luksch N. (2019). Preparation of Imidazo[5,1-b]thiazole Derivatives as Inhibitors of Indoleamine 2,3-Dioxygenase and/or Tryptophan 2,3-Dioxygenase. Patent.

[80] Nicolaou K.C., Erande R.D., Vourloumis D., Pulukuri K.K., Rigol S. (2019). Preparation of Tubulysin Analogues as Anticancer Agents and Payloads for Antibody-Drug Conjugates Useful for Treatment of Cancer. Patent.

[81] Saiah E., Vlasuk G. (2018). Preparation of Amino Acid as Modulators of Sestrin-Gator Interaction. Patent.

[82] Wang S., Zhou H., Lu J., Liu L., Sun Y., Bernard D. (2018). Preparation of Small Molecule DCN1 Inhibitors and Therapeutic Methods Using the Same. Patent.

[83] Bacon E.M., Chin E., Cottell J.J., Katana A.A., Kato D., Link J.O., Shapiro N., Trejo M., Teresa A., Yang Z.Y. (2018). Preparation of Azapeptide Atazanavir Analogs Useful for Treating HIV Infections. Patent.

[84] Röschenthaler G.V., Kukhar V.P., Kulik I.B., Belik M.Y., Sorochinsky A.E., Rusanov E.B., Soloshonok V.A. (2012). Asymmetric synthesis of phosphonotrifluoroalanine and its derivatives using N-tert-butanesulfinyl imine derived from fluoral. Tetrahedron Lett..

[85] Turcheniuk K.V., Poliashko K.O., Kukhar V.P., Rozhenko A.B., Soloshonok V.A., Sorochinsky A.E. (2012). Efficient asymmetric synthesis of trifluoromethylated β-aminophosphonates and their incorporation into dipeptides. Chem. Commun..

[86] Soloshonok V.A., Belokon Y.N., Kuzmina N.A., Maleev V.I., Svistunova N.Y., Solodenko V.A., Kukhar V.P. (1992). Asymmetric Synthesis of Phosphorus Analogs of Dicarboxylic α-Amino Acids. J. Chem. Soc. Perkin Trans..

[87] Qiu W., Gu X., Soloshonok V.A., Carducci M.D., Hruby V.J. (2001). Stereoselective Synthesis of Conformationally Constrained Reverse Turn Dipeptide Mimetics. Tetrahedron Lett..

[88] Soloshonok V.A., Sorochinsky A.E. (2010). Practical Methods for the Synthesis of Symmetrically α-Disubstituted-α-Amino Acids. Synthesis.

[89] Soloshonok V.A., Ohkura H., Yasumoto M. (2006). Operationally Convenient Asymmetric Synthesis of (*S*)- and (*R*)-3-Amino-4,4,4-trifluorobutanoic Acid. Part II: Enantioselective Biomimetic Transamination of 4,4,4-Trifluoro-3-oxo-*N*-[(*R*)-1-phenylethyl)butanamide. J. Fluor. Chem..

[90] Bravo P., Farina A., Kukhar V.P., Markovsky A.L., Meille S.V., Soloshonok V.A., Sorochinsky A.E., Viani F., Zanda M., Zappala C. (1997). Stereoselective Additions of α-Lithiated Alkyl *p*-Tolylsulfoxides to *N*-PMP Fluoroalkyl Aldimines. An Efficient Approach to Enantiomerically Pure Fluoro-Amino Compounds. J. Org. Chem..

[91] Soloshonok V.A., Kukhar V.P. (1996). Biomimetic Base-Catalyzed [1,3]-Proton Shift Reaction. A Practical Synthesis of β-Fluoroalkyl-Amino Acids. Tetrahedron.

[92] Han J., Nelson D.J., Sorochinsky A.E., Soloshonok V.A. (2011). Self-Disproportionation of Enantiomers via Sublimation; New and Truly Green Dimension in Optical Purification. Curr. Org. Synth..

[93] Sorochinsky A.E., Katagiri T., Ono T., Wzorek A., Aceña J.L., Soloshonok V.A. (2013). Optical purifications via Self-Disproportionation of Enantiomers by achiral chromatography; Case study of a series of α-CF_3_-containing secondary alcohols. Chirality.

[94] Sorochinsky A.E., Aceña J.L., Soloshonok V.A. (2013). Self-Disproportionation of Enantiomers of Chiral, Non-Racemic Fluoroorganic Compounds: Role of Fluorine as Enabling Element. Synthesis.

[95] Han J., Wzorek A., Kwiatkowska M., Soloshonok V.A., Klika K.D. (2019). The self-disproportionation of enantiomers (SDE) of amino acids and their derivatives. Amino Acids.

[96] Suzuki Y., Han J., Kitagawa O., Aceña J.L., Klika K.D., Soloshonok V.A. (2015). A comprehensive examination of the self-disproportionation of enantiomers (SDE) of chiral amides via achiral, laboratory-routine, gravity-driven column chromatography. RSC Adv..

[97] Han J., Kitagawa O., Wzorek A., Klika K.D., Soloshonok V.A. (2018). The self-disproportionation of enantiomers (SDE): A menace or an opportunity?. Chem. Sci..

[98] Han J., Soloshonok V.A., Klika K.D., Drabowicz J., Wzorek A. (2018). Chiral sulfoxides: Advances in asymmetric synthesis and problems with the accurate determination of the stereochemical outcome. Chem. Soc. Rev..

[99] Wang Y., Song X., Wang J., Moriwaki H., Soloshonok V.A., Liu H. (2017). Recent approaches for asymmetric synthesis of a-amino acids via homologation of Ni(II) complexes. Amino Acids.

[100] Aceña J.L., Sorochinsky A.E., Soloshonok V. (2014). Asymmetric synthesis of -amino acids via homologation of Ni(II) complexes of glycine Schiff bases. Part 3: Michael addition reactions and miscellaneous transformations. Amino Acids.

[101] Sorochinsky A.E., Aceña J.L., Moriwaki H., Sato T., Soloshonok V.A. (2013). Asymmetric synthesis of -amino acids via homologation of Ni(II) complexes of glycine Schiff bases. Part 2: Aldol, Mannich addition reactions, deracemization and (*S*) to (*R*) interconversion of -amino acids. Amino Acids.

[102] Sorochinsky A.E., Aceña J.L., Moriwaki H., Sato T., Soloshonok V.A. (2013). Asymmetric synthesis of α-amino acids via homologation of Ni(II) complexes of glycine Schiff bases; Part 1: Alkyl halide alkylations. Amino Acids.

[103] Ellis T.K., Ueki H., Yamada T., Ohfune Y., Soloshonok V.A. (2006). The Design, Synthesis and Evaluation of a New Generation of Modular Nucleophilic Glycine Equivalents for the Efficient Synthesis of Sterically Constrained-Amino Acids. J. Org. Chem..

[104] Soloshonok V.A., Ueki H., Ellis T.K., Yamada T., Ohfune Y. (2005). Application of Modular Nucleophilic Glycine Equivalents for Truly Practical Asymmetric Synthesis of -Substituted Pyroglutamic Acids. Tetrahedron Lett..

[105] Takeda R., Kawamura A., Kawashima A., Sato T., Moriwaki H., Izawa K., Akaji K., Wang S., Liu H., Aceña J.L. (2014). Chemical Dynamic Kinetic Resolution and (*S*)/(*R*)-Interconversion of Unprotected α-Amino Acids. Angew. Chem. Int. Ed..

[106] Jörres M., Aceña J.L., Soloshonok V.A., Bolm C. (2015). Asymmetric Carbon-Carbon Bond Formations under Solvent-Less Conditions in Ball Mills. ChemCatChem.

[107] Jörres M., Chen X., Aceña J.L., Merkens C., Bolm C., Liu H., Soloshonok V.A. (2014). Asymmetric Synthesis of a-Amino Acids under Operationally Convenient Conditions. Adv. Synth. Catal..

[108] Takeda R., Kawamura A., Kawashima A., Sato T., Moriwaki H., Izawa K., Abe H., Soloshonok V.A. (2018). Second-order asymmetric transformation and its application for the practical synthesis of α-amino acids. Org. Biomol. Chem..

[109] Takeda R., Kawamura A., Yamamoto J., Sato T., Moriwaki H., Izawa K., Abe H., Soloshonok V.A. (2018). Tandem alkylation—Second-order asymmetric transformation protocol for preparation of phenylalanine-type tailor-made α-amino acids. ACS Omega.

[110] Han J., Takeda R., Sato T., Moriwaki H., Abe H., Izawa K., Soloshonok V.A. (2019). Optical Resolution of Rimantadine. Molecules.

[111] Nian Y., Wang J., Zhou S., Wang S., Moriwaki H., Kawashima A., Soloshonok V.A., Liu H. (2015). Recyclable Ligands for the Non-Enzymatic Dynamic Kinetic Resolution of Challenging α-Amino Acids. Angew. Chem. Int. Ed..

[112] Romoff T.T., Palmer A.B., Mansour N., Creighton C.J., Miwa T., Ejima Y., Moriwaki H., Soloshonok V.A. (2017). Scale-up Synthesis of (R)- and (S)-N-(2-benzoyl-4-chlorophenyl)-1-(3,4-dichlorobenzyl)pyrrolidine-2-carboxamide hydrochloride, a Versatile Reagent for Preparation of Tailor-made α- and β-Amino Acids in Enantiomerically Pure Form. Org. Process Res. Dev..

[113] Soloshonok V.A., Ellis T.K., Ueki H., Ono T. (2009). Resolution/Deracemization of Chiral-Amino Acids Using Resolving Reagents with Flexible Stereogenic Centers. J. Am. Chem. Soc..

[114] Sorochinsky A.E., Ueki H., Aceña J.L., Ellis T.K., Moriwaki H., Soloshonok V.A. (2013). Chemical approach for interconversion of (*S*)- and (*R*)-α-amino acids. Org. Biomol. Chem..

[115] Sorochinsky A.E., Ueki H., Aceña J.L., Ellis T.K., Moriwaki H., Sato T., Soloshonok V.A. (2013). Chemical deracemization and (*S*) to (*R*) interconversion of some fluorine-containing α-amino acids. J. Fluor. Chem..

[116] Zhou S., Wang J., Chen X., Aceña J.L., Soloshonok V.A., Liu H. (2014). Chemical Kinetic Resolution of Unprotected-Substituted-Amino Acids Using Recyclable Chiral Ligands. Angew. Chem. Int. Ed..

[117] Ellis T.K., Hochla V.M., Soloshonok V.A. (2003). Efficient Synthesis of 2-Aminoindane-2-Carboxylic Acid via Dialkylation of Nucleophilic Glycine Equivalent. J. Org. Chem..

[118] Ellis T.K., Martin C.H., Tsai G.M., Ueki H., Soloshonok V.A. (2003). Efficient Synthesis of Sterically Constrained Symmetrically, -Disubstituted-Amino Acids under Operationally Convenient Conditions. J. Org. Chem..

[119] Soloshonok V.A., Avilov D.V., Kukhar V.P. (1996). Highly Diastereoselective Asymmetric Aldol Reactions of Chiral Ni(II)-Complex of Glycine with Trifluoromethyl Ketones. Tetrahedron.

[120] Soloshonok V.A., Avilov D.V., Kukhar V.P. (1996). Asymmetric Aldol Reactions of Trifluoromethyl Ketones with a Chiral Ni(II) Complex of Glycine: Stereocontrolling Effect of the Trifluoromethyl Group. Tetrahedron.

[121] Kawamura A., Moriwaki H., Röschenthaler G.V., Kawada K., Aceña J.L., Soloshonok V.A. (2015). Synthesis of (2*S*,3*S*)-β-(trifluoromethyl)-α,β-diamino acid by Mannich addition of glycine Schiff base Ni(II) complexes to *N*-*tert*-butylsulfinyl-3,3,3-trifluoroacetaldimine. J. Fluor. Chem..

[122] Soloshonok V.A., Avilov D.V., Kukhar V.P., Meervelt L.V., Mischenko N. (1997). Highly Diastereoselective aza-Aldol Reactions of a Chiral Ni(II) Complex of Glycine with Imines. An Efficient Asymmetric Approach to 3-Perfluoroalkyl-2,3-Diamino Acids. Tetrahedron Lett..

[123] Soloshonok V.A., Cai C., Hruby V.J. (2000). (*S*)- or (*R*)-*N*-(*E*-enoyl)-4-phenyl-1,3-oxazolidin-2-ones: Ideal Michael Acceptors to Afford a Virtually Complete Control of Simple and Face Diastereoselectivity in Addition Reactions with Glycine Derivatives. Org. Lett..

[124] Soloshonok V.A., Cai C., Hruby V.J., Meervelt L.V., Yamazaki T. (2000). Rational Design of Highly Diastereoselective, Organic Base-Catalyzed, Room Temperature Michael Addition Reactions. J. Org. Chem..

[125] Yamada T., Okada T., Sakaguchi K., Ohfune Y., Ueki H., Soloshonok V.A. (2006). Efficient Asymmetric Synthesis of Novel 4-Substituted and Configurationally Stable Analogs of Thalidomide. Org. Lett..

[126] Kawashima A., Xie C., Mei H., Takeda R., Kawamura A., Sato T., Moriwaki H., Izawa K., Han J., Aceña J.L. (2015). Asymmetric synthesis of (1*R*,2*S*)-1-amino-2-vinylcyclopropanecarboxylic acid by sequential S_N_2–S_N_2′ dialkylation of (*R*)-*N*-(benzyl)proline-derived glycine Schiff base Ni(II) complex. RSC Adv..

[127] Kawashima A., Shu S., Takeda R., Kawamura A., Sato T., Moriwaki H., Wang J., Izawa K., Aceña J.L., Soloshonok V.A. (2016). Advanced asymmetric synthesis of (1*R*,2*S*)-1-amino-2-vinylcyclopropanecarboxylic acid by alkylation/cyclization of newly designed axially chiral Ni(II) complex of glycine Schiff base. Amino Acids.

[128] Wang J., Lin D., Zhou S., Soloshonok V.A., Liu H. (2011). Asymmetric Synthesis of Sterically Constrained Linear Trifluoromethyl Containing Amino Acids via Alkylation of Chiral Equivalents of Nucleophilic Glycine and Alanine. J. Org. Chem..

[129] Tang X., Soloshonok V.A., Hruby V.J. (2000). Convenient Asymmetric Synthesis of Enantiomerically Pure 2’,6’-Dimethyltyrosine (DMT) via Alkylation of Chiral Nucleophilic Glycine Equivalent. Tetrahedron.

[130] Soloshonok V.A., Ono T., Ueki H., Vanthuyne N., Balaban T.S., Bürck J., Fliegl H., Klopper W., Naubron J.V., Tam T.T. (2010). Ridge-tile-like chiral topology: Synthesis, resolution and complete chiroptical characterization of enantiomers of edge-sharing binuclear square planar complexes of Ni(II) bearing achiral ligands. J. Am. Chem. Soc..

[131] Soloshonok V.A., Ueki H. (2007). Design, Synthesis and Characterization of Binuclear Ni(II) Complexes with Inherent Helical Chirality. J. Am. Chem. Soc..

