# Supplementary material for: Preparative Method for Asymmetric Synthesis of (S)-2-Amino-4,4,4-trifluorobutanoic Acid
Source: Molecules. 2019 Dec 10;24(24):4521. doi: 10.3390/molecules24244521 (PMC6943542; doi:10.3390/molecules24244521)
Supplement: Supplementary file 1 [file molecules-24-04521-s001.pdf]

molecules

MDPI

Supporting Information

Preparative Method for Asymmetric Synthesis of (*S*)-2-Amino-4,4,4-trifluorobutanoic Acid Derivatives

Jianlin Han, Ryosuke Takeda, Xinyi Liu, Hiroyuki Konno, Hidenori Abe *, Takahiro Hiramatsu, Hiroki Moriwaki * and Vadim A. Soloshonok *

1. General procedure……………………………………………………………………...S2

2. HPLC analysis of alkylation reaction of (*S*)-6 with 1,1,1-trifluoro-2-iodoethane………...S4

3. Purification and ^{1}H NMR of (*S*)-12……………………………………..………………S5

4. HPLC analyses of Fmoc-protection of (*S*)-12……………………..……..………………S6

5. Analytical data of (*S*)-12………………………………………..………………………S7

6. HPLC analysis conditions and spectra of 7 and 12…………………………………………S8

1. General Procedure

*1.1. Alkylation of (S)-**6** with ICH_2CF_3*

For large-scale preparation, the (*S*)-**6** the procedure described in [1] was followed.

To a 5000 mL four-necked flask was added *N*,*N*-dimethyl-formamide (DMF) (2250 mL) under nitrogen atmosphere. Stirring was continued for 50 min with nitrogen flow. Then, the Ni–glycine complex (*S*)-**6** (250.0 g, 1.0 equiv) together with DMF (100 mL), 1,1,1-trifluoro-2-iodoethane (91.61 g, 1.05 equiv) together with DMF (50 mL), and KOH (25.42 g, 1.05 equiv) in MeOH (225 mL) together with DMF (100 mL) were added to the flask. The mixture was stirred at 20~35 °C under a nitrogen atmosphere for 1 h. After that, H_2O (750 mL, 3 v) was added into the mixture and stirred for 1 h at 20~40 °C and precipitate was formed gradually. Then, H_2O (500 mL, 2 v) was added and stirred at the same temperature for 1 h. The precipitate was filtered and washed with H_2O (500 mL, 2 v), then dried by air for 19.5 h to give the product (*S*)(2*S*)-**7** (228.50 g, 80.4% yield, 97.2 area%, >99% de).

*1.2. Disassembly of Major Diastereomer (S)(2S)-**7** and Preparation of (S)-**12***

To a 2000 mL four-necked flask was added dimethoxyethane (DME) (400 mL), Ni complex (*S*,2*S*)-**7** (220 g, 1.0 equiv) together with DME (30 mL) and HCl (6N, 268 mL, 5.0 equiv) together with DME (10 mL). The mixture was heated to 40~50 °C and stirred at this temperature for 1 h. After that, the solution was changed to a green suspension and was cooled to 20~40 °C. H_2O (440 mL, 2 v) was added and the mixture was stirred at 20~40 °C for 1.5 h. The precipitate was filtered and washed with DME (88 mL, 0.4 v), 6N HCl (59 mL, 0.27 v), H_2O (293 mL, 1.33 v), then H_2O (220 mL, 1 v). The green filtrate was collected to give a solution of (*S*)-**1** (ca. 1500 mL).

To a 3000 mL 4-necked flask containing the above green filtrate was added ethylenediaminetetraacetic acid disodium salt hydrate (122.2 g, 1.02 equiv). With stirring, 48% NaOH (138 g, 5.2 equiv), Na_2CO_3 (1.3 equiv 44.35 g), Fmoc-OSu (1 equiv, 108.57 g), and acetonitrile (200 mL, 4 v) were added. The resulted mixture was stirred at room temperature for 4 h and acetonitrile was removed. Then, EtOAc (180 mL) and 6N HCl (70 mL, 4 eq.) were added and the phases were separated. The water layer was extracted with ethyl acetate (100 mL), and the combined organic layer was washed with water (100 mL). The combined organic solution was dried with Na_2SO_4 (30 g), and then the filtrate was concentrated to 400 mL. The resulted solution was heated to 50~60 °C, and toluene (400 mL) was added. After that, it was concentrated until 400 mL, and EtOAc (100 mL) was added. Finally, toluene (400 mL) was added and then the solution was concentrated, followed by addition of additional toluene (400 mL) until 800 mL with stirring slowly at 20~35 °C. The precipitate was filtered, washed with toluene (160 mL), and dried in vacuo at 50 °C for 19 h to afford (*S*)-**12** (99.5 g, 81.5% yield 98.4% ee, a white powder).

2. HPLC Analysis of Alkylation Reaction of (*S*)-6 with 1,1,1-Trifluoro-2-iodoethane

Exp. #	Sample	RT/min, Peak Assign										Comments
		15.6	16.8	17.5	18.0	19.3	20.1	20.7	21.5	25.9	27.2	
		QNZ	Oxidized	Hydroxy	CBPBG	Major	Minor	bis-alkyl	uk	uk	Ligand	
1391-065 for 30 g	10 min	0.26	0.29	1.41	1.01	78.43	1.89	3.54	0.36	0.47	2.72	95.3% de [b]
	1 h	0.28	0.33	0.52	0.74	86.38	2.08	6.04	0.38	0.40	1.06	95.3% de
	solid	n.d.	0.21	n.d.	0.18	97.66	n.d. [a]	1.22	0.39	0.10	0.20	>99% de
	ML	5.97	1.54	2.49	3.79	15.17	0.86	28.49	n.d.	1.62	20.03	-
1391-069 1st batch for 170 g	15 min	0.27	0.72	1.67	0.82	79.32	1.94	4.23	0.32	0.35	1.52	95.2% de
	1 h	n.d.	0.35	1.12	1.16	86.42	2.20	5.87	0.43	0.33	0.85	95.0% de
	solid	n.d.	0.20	0.25	0.31	97.13	0.02	1.20	0.44	0.08	0.19	99.9% de
	ML	n.d.	1.80	3.99	8.60	20.30	1.26	31.83	n.d.	1.34	20.14	-
1391-070 2nd batch for 170 g	15 min	n.d.	1.23	0.24	0.73	85.44	1.07	4.16	0.32	0.38	1.71	97.5% de
	1 h	n.d.	0.33	0.23	1.17	87.75	1.15	5.50	0.34	0.29	1.05	97.4% de
	solid	n.d.	0.18	n.d.	0.41	97.16	n.d.	1.22	n.d.	n.d.	0.41	>99% de
	ML	n.d.	1.72	0.75	10.93	18.05	1.32	29.31	n.d.	1.16	25.66	-

[a] n.d. = not detected. [b] de = diastereomeric excess

3. Purification and ^{1}H–NMR of (*S*)-12

Exp. # 1391-068 Purification of (*S*)-**12.**

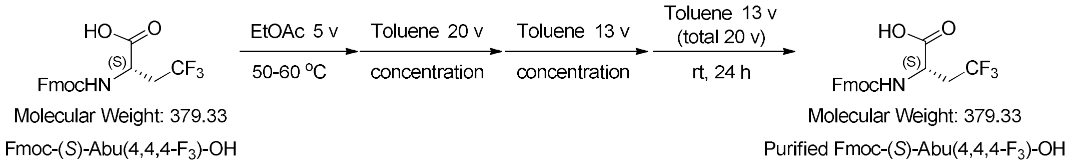

Exp. #	Scale	Conditions	Results
1391-068	Fmoc-(*S*)-Abu(4,4,4-F_3)-OH 35 g 98.5 area%, 98.9% ee	EtOAc (5 v, 175 mL), Toluene (20+13+13+5 v, 700+455+455+175 mL) room temperature (rt), 24 h	34.27 g, 97.9% yield 98.8 area%, 99.0% ee (enantiomeri c excess)

^{1}H-NMR Spectrum of (*S*)-12 Dry Solid in MeOH-*d_4* (Lot # 1391-06835)

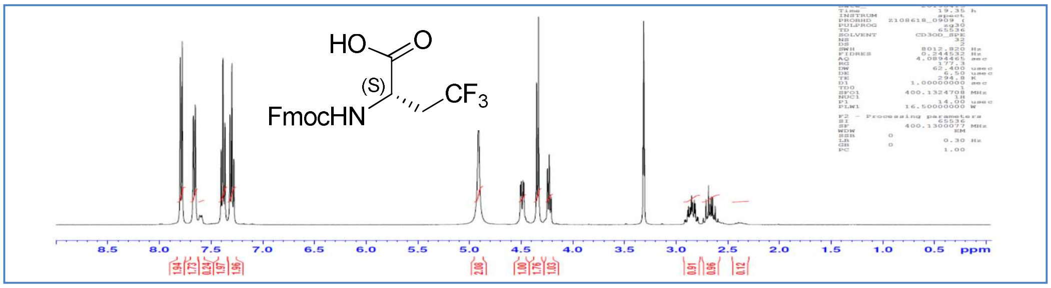

4. HPLC Analyses of Fmoc-Protection of (*S*)-12

Exp. #	Sample	RT/min, Peak Assign									Comments
		15.3	15.4	16.0	16.8	17.7	19.0	19.8	21.0	27.2	
		Fmoc-Gly	Fmoc-β-Ala	uk	Fmoc-OSu	Fmoc-AA Fmoc-OSu Toluene	uk	bis-alkyl	DBF	Ligand	
1391-067	crude	n.d.	0.43	0.02	n.d.	98.53	0.40	0.07	0.02	0.15	98.9% ee
1391-068	purified	n.d.	0.23	n.d.	n.d.	98.80	n.d.	n.d.	n.d.	0.07	99.0% ee
1391-075	1st batch	0.29	0.22	n.d.	0.07	98.72	0.23	0.09	0.11	0.02	98.8% ee
1391-077	2nd batch	0.39	0.14	n.d.	0.13	97.89	0.67	0.15	0.09	0.05	98.4% ee
1391-078	purified	0.34	0.14	n.d.	0.03	99.32	0.05	n.d.	0.03	0.07	99.1% ee
	recovery	0.47	0.60	n.d.	0.46	96.17	1.22	0.26	0.40	0.09	>99.9% ee
	reprocess	0.49	0.57	n.d.	0.42	97.03	0.26	n.d.	0.37	0.37	100% ee

5. Analytical Data of (*S*)-12

	30 g Synthesis		170 g Synthesis				
	**Fmoc-Protection**	**Purification**	**Fmoc-Protection 1st batch**	**Fmoc-Protection 2nd batch**	**Purification Combined Batch**	**Recovery from ML**	**Reprocess**
Lot #	1391-06755	1391-06835	1391-07554	1391-07754	1391-07835	1391-07840	1391-07854
amount	35.32 g	34.27 g	92.66 g	99.50 g	163.20 g	17.13 g	16.25 g
yield	(87.2%)	97.9%	(75.9%)	(81.5%)	85.5%	9.0%	8.5% (95.6%)
HPLC purity	98.53 area%	98.80 area%	98.7 area%	97.9 area%	99.32 area%	96.16 area%	97.03 area%
optical purity	98.9% ee	99.0% ee	98.8% ee	98.4% ee	99.1% ee	>99.9% ee	100% ee
impurity	Fmoc-Gly: n.d. Fmoc-β-Ala: 0.43% 19.0 min: 0.40% (20.3 min: 0.05%)	Fmoc-Gly: n.d. Fmoc-β-Ala: 0.23% 19.0 min: n.d. (20.3 min: 0.50%)	Fmoc-Gly: n.d. Fmoc-β-Ala: 0.43% 19.0 min: 0.40% (20.3 min: 0.05%)	Fmoc-Gly: n.d. Fmoc-β-Ala: 0.43% 19.0 min: 0.40% (20.3 min: 0.05%)	Fmoc-Gly: 0.34% Fmoc-β-Ala: 0.14% 19.0 min: 0.05% (20.3 min: n.d.)	Fmoc-Gly: 0.48% Fmoc-β-Ala: 0.58% 19.0 min: 1.22% (20.3 min: n.d.)	Fmoc-Gly: 0.49% Fmoc-β-Ala: 0.57% 19.0 min: 0.26% (20.3 min: n.d.)

6. HPLC Analysis Conditions and Spectra of 7 and 12

6.1. HPLC Conditions (achiral)

Column: Inertsil ODS-3, S-3 μm, ϕ4.6 × 150 mm
Solvent A: 0.01 M $HCOONH_4$ in 0.1% HCOOH aq
Solvent B: acetonitrile
Flow rate: 1.0 mL/min; column temperature: 30 °C
UV: 254 nm

Time(min)	0.0	15.00	20.00	23.00	30.00	30.01	37.00
Solvent A (%)	95	20	20	0	0	95	95
Solvent B (%)	5	80	80	100	100	5	5

*6.2. Chiral HPLC Conditions for (S)-**12** (200 min Method)*

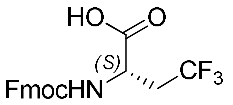

Molecular Weight: 379.3298
Fmoc-(*S*)-Abu(4,4,4-F_3)

Column: CHIRALPAK IC (DAICEL CHEMICAL), S-5 μm, ϕ 4.6 × 150 mm
Solvent A: 0.1% H_3PO_4 in H_2O
Solvent B: 0.1% H_3PO_4 in acetonitrile
Flow rate: 0.6 mL/min.
Column temperature: 20 °C
UV: 254 nm
Gradient: Isocratic (A:B = 72:28), 200 min

*6.3. Chiral HPLC Chart of (S)-**12** (98.4% ee, Lot # 1391-07754)*

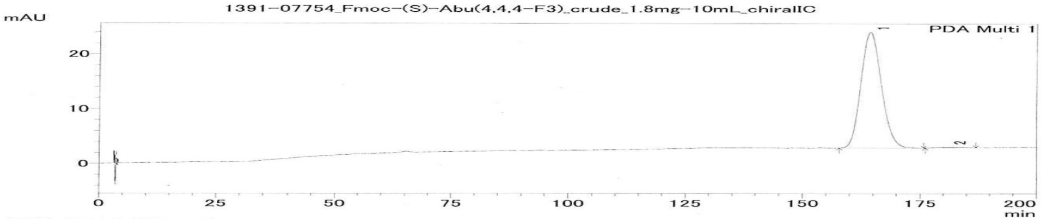

*6.4. Chiral HPLC Chart of (S)-**12** (99.1% ee, Lot # 1391-07835)*

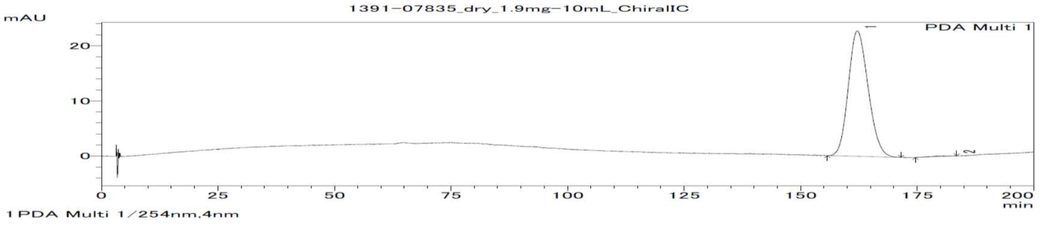

6.5. Chiral HPLC Chart of **12** *((S)- and (R)- Mixture)*

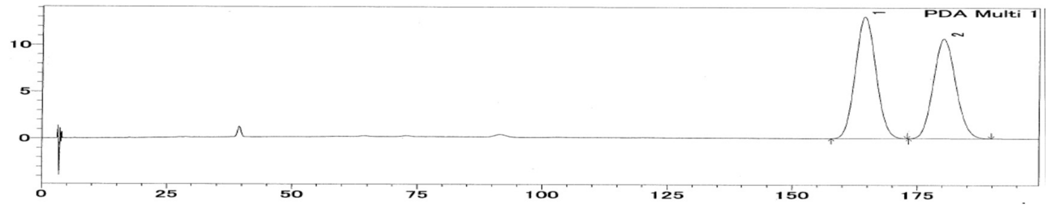

*6.6. Chiral HPLC Analysis of (S,S)-***7**

The CHIRALPAK IA-e column was used for the analysis of optical purity.

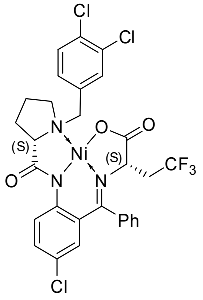

Molecular Weight: 683.56
(*S*,*S*)-CBPBAbu(4,4,4-F_3)

6.6.1. Chiral HPLC Conditions for (*S*,*S*)-**7**

Column: CHIRALPAK IA-3 (DAICEL CHEMICAL), S-3 μm, ϕ 4.6 × 250 mm
Solvent A: MeOH
Solvent B: H_2O
Gradient: A:B = 90:10, 30 min (isocratic)
Flow rate: 0.5 mL/min.
Column temperature: 40 °C
UV: 254 nm

6.6.2. Chiral HPLC Chart of CBPBAbu(4,4,4-F_3) ((S,S)- and (R,R)- mixture)

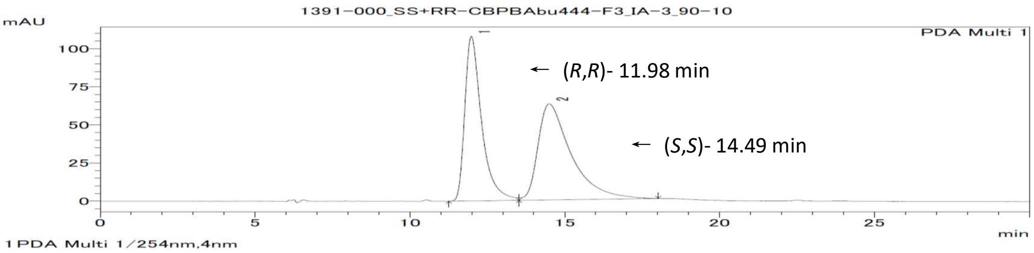

6.6.3. Chiral HPLC Chart of (*S*,*S*)-CBPBAbu(4,4,4-F_3) (100% ee, Lot # 1391-07039, 2.1 mg)

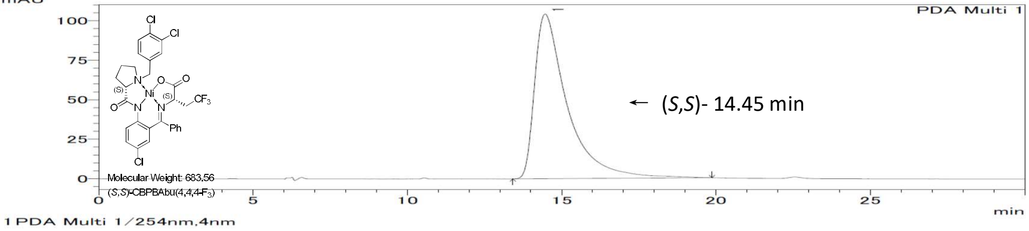

6.6.4. Chiral HPLC Chart of (*R*,*R*)-CBPBAbu(4,4,4-F_3) (99.8% ee, Lot # 1423-05615, 2.4 mg)

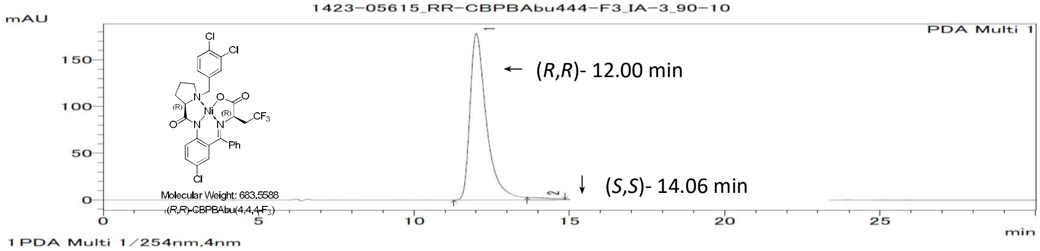

References

1. Ueki, H.; Ellis, T.K.; Martin, C.H.; Bolene, S.B.; Boettiger, T.U.; Soloshonok, V.A. Improved Synthesis of Proline Derived Ni(II)-Complexes of Glycine, a Versatile Chiral Equivalents of Nucleophilic Glycine for General Asymmetric Synthesis of α-Amino Acids, *J. Org. Chem.* **2003**, *68*, 7104–7107.
